# Meeting the Calls of Necessity

**Published:** 1907-03

**Authors:** T. C. Gibson

**Affiliations:** Forsyth, Ga.


### The American Journal of Dental Science

The Official Organ of the Florida, Georgia and South Carolina State Dental Societies

**Third Series**

VOL. XXXVIII MARCH, 1907 NO. 3

EDITORS

WM. GIRD BEECROFT, D. D. S., Madison. Wis. B. H. TEAGUE, D. D. S., Aiken, S. C.

Published on the 10th day of each month.
Subscription price $1.00 per year.
ENTERED AS SECOND-CLASS MATTER, MADISON, WIS.

Address all communications, both business and editorial, to
**The Dental Publishing Company, Madison, Wisconsin**

### MEETING THE CALLS OF NECESSITY.

By Dr. T. C. Gibson, Forsyth, Ga.

When a child I often dreamed of tasks seemingly impossible of accomplishment, and as I looked and considered, they were performed as if by a magic wand waved above them. Sometimes I saw in my dreams skeins of thread so knotted and tangled that they could not be more intricately commingled, and these were to be straightened and brought into order. My heart would sink within me as I considered the task, but these too, yielded quite readily to properly directed thought and effort. Seemingly some unseen hand would direct as with patience, I set about the apparently impossible task, difficulties and intricacies dissolving and disappearing.

In my youth I set many points, the attainment of which I expected to mark the culmination of my efforts, the beginning of a life of ease and manhood free from further efforts and worry, free from further difficulties to overcome; but alas, the nearing of these stated points but removed the object of final attainment further, and yet further away in the future.

When at the age of twenty-two my father said, "Son, your education is completed; yonder is a farm, possess it, and live the life of a Christian man." Contented on the farm, I began life for myself. For several years I lived upon the farm, distantly removed from the conveniences of modern living, as represented in cities and towns, with their railroads, daily express, daily mails, etc., comparatively contented with the hard tasks of him who would best succeed in agricultural pursuits. My reading was confined almost exclusively to the perusal of a few antiquated books, the gift of my father.

As time passed on my friends and neighbors called on me to fill this position and that position. My country called and I responded. The railroad came, the town and postoffice located near me, and I had to move on; circumstances commanded me to go forward. Married at the age of thirty-six years, I thought "I'm settled now," and for a time I was retired. But the time came when I felt the necessity of again taking my books and returning to the school-room. At the age of forty I took my place among the student body. In due time my college conferred upon me her diploma, bidding me enjoy all the privileges of a graduated D. D. S., and reflect as much credit as I might be able upon my profession and my Alma Mater. Again I went forth to new pursuits, locating in a thriving little city. I began to enjoy my acquirements, and for two years I labored and strove to do my best, in an humble way, until I received an earnest solicitation from one of my most highly esteemed friends, a gifted member of this Association, my former teacher to identify myself with your body. This I did, becoming a member of this body at the meeting held in the City of Macon, Ga. I came, I joined, I participated. My work in this body is recorded in your minutes. During my college career I said to my fellow students, you may have the honors, I have a wife and family, all I want is to learn dentistry, and it is not my fault if both the college and my class sought to do me honor. I call upon you, gentlemen of this Society, to bear me witness this day that I have not sought advancement at your hands. I have only tried to do what I conceived to be my duty toward the upbuilding and improvement of this body.

When I have read a paper or made a demonstration, I have always felt my weakness. I have always conceived that others could cover the ground better than I, but seemingly there has been a demand upon me to do this or that, and I have simply done my best. When I have written a paper and tried to read its contents, I have always felt, how commonplace, how weak; surely this will not do to read before that educated audience. Trembling and in doubt I have stood before you, as I indeed this day stand, and read to you my poor thoughts.

When at my office, a patient presents himself, and I find difficult work to be done, and he asks, "Can these conditions be corrected, these teeth saved?" I am in the habit of replying, "Anything can be done provided we have the skill to do it."

Of course there are many points to consider, such as, will the patient give the proper attention and sufficient time to the engagement? will he be willing to continue treatment sufficiently long for us to correct chronic conditions? and, lastly, will he be willing to pay us a reasonable fee for our services? These questions being decided in the affirmative, I begin usually with the least difficult work and proceed gradually to that which is more difficult, until the work is reduced to one or more teeth. The difficult features of the work gradually disappear, and I usually find the work not near so difficult to accomplish satisfactorily as at first I had expected.

Thus difficulties vanish before him of skill and the courage to attack them.

I would not advise that we approach our work without thought or plans, and I have the deepest pity for him who is satisfied with his attainments.

We should ever strive to broaden our knowledge, to heighten our attainments and perfect our skill, and no man can do himself, his patients, or his profession any degree of justice, who, becoming self-satisfied, neglects to do these things. To repeat an old, but ever true adage, "Necessity is the mother of invention," and so sure as this is true, necessity has evolved our present high state of enlightened advancement and civilization, and as a part of this, stands our profession of dentistry. It leads every worker who rationally pursues his labors, and he alone is best qualified to understand and meet her calls who has appropriated and embodied within himself, the most truth on the given line.

We must understand nature's ways to work in harmony therewith.

